# Synthesis of Bamboo-like Multiwall Carbon Nanotube–Poly(Acrylic Acid-co-Itaconic Acid)/NaOH Composite Hydrogel and its Potential Application for Electrochemical Detection of Cadmium(II)

**DOI:** 10.3390/bios10100147

**Published:** 2020-10-19

**Authors:** Luis F. Chazaro-Ruiz, Miguel Olvera-Sosa, Gabriela Vidal, J. Rene Rangel-Mendez, Gabriela Palestino, Fatima Perez, Wei Zhang

**Affiliations:** 1 División de Ciencias Ambientales, Instituto Potosino de Investigación Científica y Tecnológica, A.C. (IPICYT), Camino a la Presa San José 2055, Lomas 4ª Sección, San Luis Potosí C.P. 78216, SLP, Mexico; f_ivanka@hotmail.com (G.V.); rene@ipicyt.edu.mx (J.R.R.-M.); 2 Facultad de Ciencias Químicas, Universidad Autónoma de San Luis Potosí, Manuel Nava No. 6, San Luis Potosí C.P. 78210, Mexico; miguel_olvera@outlook.com (M.O.-S.); palestinogabriela@uaslp.mx (G.P.); 3 Sección de Biotecnología, Centro de Investigación en Ciencias de la Salud y Biomedicina, Universidad Autónoma de San Luis Potosí, Avenida Sierra Leona 550, Lomas Segunda Sección, San Luis Potosí C.P. 78210, Mexico; 4 CONACYT—Instituto Potosino de Investigación Científica y Tecnológica, A.C. (IPICYT), Camino a la Presa San José 2055, Lomas 4ª Sección, San Luis Potosí C.P. 78216, SLP, Mexico; fatima.perez@ipicyt.edu.mx; 5 College of Engineering, Bay Campus, Swansea University, Swansea SA1 8EN, UK

**Keywords:** hydrogel, copolymer, carbon nanotubes, composite, carbon–paste electrode, cadmium (II) detection

## Abstract

A poly(acrylic acid-co-itaconic acid) (PAA-co-IA)/NaOH hydrogel containing bamboo-type multiwall carbon nanotubes (B-MWCNTs) doped with nitrogen (PAA-co-IA/NaOH/B-MWCNTs) was synthesized and characterized by SEM, absorption of water, point of zero charges, infrared spectroscopy, thermogravimetric analysis, and differential scanning calorimetry. The possible use of the PAA-co-IA/NaOH/B-MWCNT hydrogel as an electrode modifier and pre-concentrator agent for Cd(II) sensing purposes was then evaluated using carbon paste electrodes via differential pulse voltammetry. The presence of the B-MWCNTs in the hydrogel matrix decreased its degree of swelling, stabilized the structure of the swollen gel, and favored the detection of 3 ppb Cd(II), which is comparable to the World Health Organization’s allowable maximum value in drinking water. A calibration curve was obtained in the concentration range of 2.67 × 10^−8^ to 6.23 × 10^−7^ M (i.e., 3 and 70 ppb) to determine a limit of detection (LOD) of 19.24 μgL^−1^ and a sensitivity of 0.15 μC ppb^−1^. Also, the Zn(II), Hg(II), Pb(II) and Cu(II) ions interfered moderately on the determination of Cd(II).

## 1. Introduction

Multiwall carbon nanotubes (MWCNTs) have been used in numerous applications, including biomedicine, energy conversion and storage, as well as in the construction of nanoscale electronic and electrochemical devices such as sensors and biosensors [1]. One of the disadvantages of the MWCNTs is their poor solubility in all solvents, which limits their real application. Their chemical modification improves their solubilization and chemical functionality [2,3,4], but this treatment usually destroys their carbon wall, and thus greatly changes their desirable physical and chemical properties. To improve their manipulation and performance on mechanical and chemical operations, non-covalent and covalently crosslinked MWCNTs with polymeric materials have been prepared. This type of processing can rearrange them into 3D networks, which does not disturb their large π-electronic surface. The non-covalent wrapping of their surface with polyanionic materials [5], a hydrophilic non-charged polymer [6,7,8], a polyelectrolyte multilayer film [9], and the linear poly(acrylic acid) [10] have been reported. These interactions made the CNTs stable in water and preserved their electrocatalytic properties in the redox reactions. Therefore, a stable film of poly(acrylic acid)–MWCNTs exhibited excellent electrocatalytic activity in the oxidation of many important biomolecules, such as the biological coenzyme NADH [11], for developing of bioelectronic devices and biosensing applications. On the other hand, covalently crosslinked MWCNTs with polymeric chains have led to the preparation of nanocomposite hydrogels, which have better mechanical, thermal, optical, and electrical properties compared to pure hydrogels [12].

Other composites generated through the reaction of ionic polymers and metals have been successfully used as dynamic sensors, transducers, and actuators. In this regard, these ionic polymer-metal composites (IPMCs) have been studied for various applications such as integration into energy harvesting as systems [13], for vibration control [14] applications in flapping-wing micro air vehicles [15], and as biomimetic actuators [16], among others.

Regarding the bamboo-like multiwall carbon nanotubes doped with nitrogen (B-MWCNTs), these are tubes divided into small compartments, which are produced by partial substitutions of carbon atoms by nitrogen atoms as dopants. Their electrical conductivity and chemical reactivity are greatly improved due to the introduction of an unpaired electron pair per nitrogen atom [17]. Among their applications is the manufacture of semiconductors [18], and the development of electrochemical biosensors, since they can act as electrocatalysts. Also, B-MWCNTs show biocompatibility, allowing the immobilization of biomolecules without loss of their activity. They also have been used as a transducer for the detection of biomolecules [19]. Furthermore, the B-MWCNTs have been applied for the removal of metal ions such as Cr(III) [20] in their pristine form. After they received prior oxidative treatment, they were found to be more effectively used in the adsorption of Cd(II) and Pb(II) [21].

Cadmium is of environmental concern since it is a toxic element that can be present in water, air, and soil as a result of natural and anthropogenic processes [22]. The World Health Organization (WHO, 2010) provides a concentration of 0.003 mg L^−1^ as the maximum permissible limit in water for human use and consumption [23], while the Environmental Protection Agency (EPA) recommends a concentration of 0.005 mg L^−1^ [24]. However, due to the toxicity of cadmium ions, it is not only essential to detect but also to quantify them efficiently. Electrochemical methods such as stripping voltammetry is one of the most favorable techniques for the determination of heavy metal ions [25], including cadmium [26]. One study that developed electrochemical sensors for the detection of cadmium through the modification of a glassy carbon electrode with a film of MWCNT/Nafion reached a detection limit of 51 nM [27]. Another study with a film of single-walled carbon nanotubes (SWCNTs) subsequently coated with mercury and bismuth achieved a detection limit of 0.98 ppb [28]. A coating of MWCNT/Nafion/poly(1,5-diaminonaphthalene) on platinum electrodes detected 3.2 ppb [29]. In addition, modified carbon paste electrodes (CPEs) have demonstrated many advantages by being inexpensive and easy to prepare, as they do not require extensive cleaning processes [30].

In the present work, the objective was to prepare a hydrogel composite of poly(acrylic acid-co-itaconic acid) (PAA-co-IA) partially neutralized with NaOH using a radical copolymerization containing B-MWCNTs (PAA-co-IA/NaOH/B-MWCNTs) to be used as a modifier of CPEs capable of detecting Cd(II) in aqueous solution. Poly(acrylic acid) is a common polymer in the synthesis of hydrogels, due to the carboxylic acid groups of the matrix producing high swelling capabilities with an affinity for metal ions. On the other hand, itaconic acid (IA) has been used to synthesize copolymers, essentially because it can increase the number of carboxyl groups and the hydrogen bond, to improve the water-absorption and the chelating capacities of the resulting polymers. The absorption of Cu(II), Zn(II), Ni(II), Co(II), Cd(II), Pb(II), and Hg(II) ions was evaluated with PAA-co-IA hydrogels, where it was determined that the degree of swelling was proportional to the content of itaconic acid [31]. In a previous work of the group, it was demonstrated that NaOH hydrogel neutralizing was a key factor in swelling as well as in its ability to remove rust in metallic surfaces [32]. In this effort, a novel synthesis of PAA-co-IA/NaOH/B-MWCNT hydrogel was developed for potential use as an electrode modifier of Cd(II) sensing carbon paste electrodes. Characterization of the composite hydrogel was implemented for the determination of copolymer formation, morphology, and thermal properties. In general, the advantage of this method is its easy fabrication at minimal a cost and its low limit of detection (LOD) as a Cd(II)-selective sensor with a detection limit comparable to the WHO guideline values. The use of this material as a pre-concentrating agent of Cd(II) at the electrode surface was evaluated, since the metal ion can form complexes with the carboxyl and other oxygenated functional groups on the partially oxidized B-MWCNTs.

## 2. Materials and Methods

### 2.1. Chemicals

B-MWCNTs with a 10–20 nm outer diameter, 20 mm length, 95% purity, containing 5% of iron oxy(hydro)oxides, were synthesized at IPICYT Mexico, and their characterization has been previously reported [21]. The nanotubes were partially oxidized by heat treatment in thermo-gravimetric (TGA) TA Instruments Q500 High-resolution V6.7 to a temperature at which a weight loss of 10% was reached. The sample (0.01 g) was placed in a platinum container and heated up from 50 to 125 °C, at a heating rate of 5 °C/min with 60 mL/min of air used as a carrier throughout the treatment. Next, nanotubes were dispersed in the mixture of the monomers. Acrylic acid (monomer), itaconic acid (monomer), N,N′-methylenebisacrylamide (crosslinker), 2,2′-azobis (2 amidinopropane) dihydrochloride (initiator), and paraffin oil were purchased from Sigma Aldrich. Cd(NO_3_)_2_·4H_2_O and KNO_3_ were supplied by Fluka and Fermont, respectively; Pb(NO_3_)_2_, Hg(NO_3_)_2_·2H_2_O, Cu(NO_3_)_2_·3H_2_O, and Zn(NO_3_)_2_·6H_2_O from Sigma Aldrich. All materials were used as they were received. NaOH and HCl 1 M standard solutions were prepared with deionized water (18 MΩ).

### 2.2. Synthesis of PAA-co-IA/NaOH/B-MWCNTs

**Figure 1:**
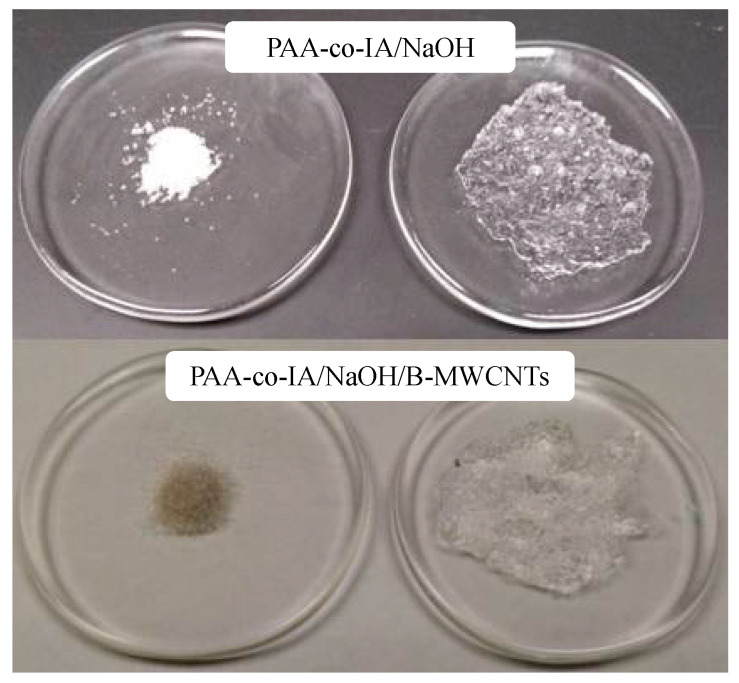
Images of dry and swollen PAA-co-IA/NaoH and PAA-co-IA/NaOH/B-MWCNTs hydrogels.

Synthesis of poly(acrylic acid-co-itaconic acid)/NaOH hydrogel (PAA-co-IA/NaOH) was carried out via free radicals. A solution mixture of acrylic acid (80 wt %) and itaconic acid (20 wt %) monomers in deionized water was prepared in a total monomer concentration at 24 wt %. The amount of initiator and crosslinker were 0.4% and 0.2% in weight, which was optimized previously [33], regarding the monomers, respectively. NaOH (7.5 wt % in respect to the monomers) was added at beginning of the reaction to assure the dissociation of the carboxylic groups. The reaction was initiated at 60 °C after the addition of the initiator and was maintained for three hours. After this time, the resulting materials were washed several times with deionized water to eliminate any residual reagents and dried at 50 °C for five days. The synthesis of the PAA-co-IA/NaOH/B-MWCNTs was carried out following the methodology described above, and the B-MWCNT were added 30 s after the monomers had been mixed. The total percentage of B-MWCNTs in the hydrogel was 0.01% relative to the total percentage of monomers. The hydrogel containing B-MWCNTs acquired a dark tone, as can be seen in Figure 1.

### 2.3. Characterization

**Physicochemical characterization.** Fourier-transform infrared (FT-IR) analysis of the MWCNTs and synthesized hydrogels was carried out using a Thermo Nicolet 6700 KBr-FT-IR in transmittance mode within a range of 600 to 4000 cm^−1^, with a 4 cm^−1^ resolution and 32 scans. For sample preparation, GO-based materials were mixed with KBr at a ratio of 1:99% (*w*/*w*) for subsequent drying at 60 °C for 48 h, then compressed into a transparent pellet for measurement.

**Morphological analysis.** The morphological analysis was carried out on an FEI Helios Nanolab 600 Dual Beam Scanning Electron Microscope (SEM) operated at 5.00 kV and 86 pA. The SEM sample was prepared by placing a drop of dilute ethanol dispersion of the composites onto a copper plate attached to an aluminum sample holder in order to evaporate the solvent at room temperature.

**Swelling capability.** The swelling capacity of PAA-co-IA/NaOH and PAA-co-IA/NaOH/MWCNT hydrogels in deionized water were gravimetrically estimated. As a first step, the dry hydrogel was powdered and then separated by filtering through a 100-mesh aluminum screen for 2 h. Then, using a nylon filter, the dried hydrogel powder was immersed in 50 mL of buffered solutions at either pH 5 or 6.6 for 16 h and weighted at different intervals of time to measure the water absorption kinetics. In the first hour of the experiment, the weights were recorded every 15 min, in the second hour every 20 min, and in the third hour every 30 min to complete the total time. Measurements were carried out in duplicate. The hydrogel percentage of water absorption (*Q*) was calculated following Equation (1).

(1) $$Q=\frac{\left(B-A\right)}{A}*100$$

where *A* and *B* are the weights of the dry polymer (g) and the swollen polymer (g), respectively [33].

**Thermal stability.** The thermal properties of hydrogels were examined by thermogravimetric analysis (TGA) and differential scanning calorimetry (DSC) using a TA Instruments Q500 TGA and a PerkinElmer DSC 8500, respectively. The samples for TGA were placed in aluminum pans and heated from room temperature to 500 °C. Nitrogen and air fluxes were kept at 20 and 40 mL/min, respectively. For DSC evaluation, samples were heated at 5 °C/min from−60 to 100 °C in a nitrogen atmosphere.

### 2.4. Electrode Preparation

The CPEs were prepared according to the methodology used by Bejarano-Jimenez et al. [32]. The dried PAA-co-IA/NaOH and PAA-co-IA/NaOH/B-MWCNT polymers were crushed to pass through a <150 mm sieve. In an agate mortar, high purity graphite powder (56 wt %), either PAA-co-IA or PAA-co-IA/B-MWCNT hydrogel (4 wt %), and paraffin oil (40 wt %) were mixed. The optimal ratio of graphite, hydrogels, and oil was obtained by studying the response of the modified CPEs using the typical reversible redox system made up of K_4_[Fe(CN)_6_] 0.01 mol L^−1^ in KCl 0.1 mol L^−1^ at pH 6 [28]. The cyclic voltammetry responses using the reversible system are shown in Supplementary Figure S1. The decrease in the current of the modified electrode responses was due to electrostatic repulsion between the negative surface charge of the hydrogels and the electroactive anion [Fe(CN)_6_]^3−^/[Fe(CN)_6_]^4−^. However, these responses demonstrated that with the hydrogel as a modifying agent it is possible to obtain an electroactive surface. The resulting pastes were packed into plastic tubes (0.15 cm diameter and 7 cm long). Electrical contact was achieved by inserting a copper wire into the packed plastic tube. The surface was renewed before each experiment by pushing an excess of paste out of the tube and polishing the new surface with filter paper.

### 2.5. Electrochemical Equipment

The electrochemical analysis was performed in a three-electrode electrochemical cell connected to a potentiostat/galvanostat model VMP3 Bio-Logic SAS coupled to EC-Lab software version 10.23. The working electrode consisted of a carbon paste modified with either PAA-co-IA/NaOH or PAA-co-IA/NaOH/B-MWCNT hydrogel. The Ag/AgCl/KCl (sat.) system was used as a reference electrode and a glassy carbon rod as the auxiliary electrode.

### 2.6. Electroanalytical Procedure

The electroanalytical performance of the PAA-co-IA/NaOH/MWCNT hydrogel was studied using differential pulse voltammetry (DPV) in 1 × 10^−3^ M Cd(II) and 0.1 M KNO_3_ electrolytic solution at pH 5. The solutions were deoxygenated with argon for 10 min before each test. The detection procedure was performed following the next stages: (1) The surface of the modified carbon paste electrode was pre-treated before use in the detection of Cd(II) by applying a cathodic potential of −1.0 V for 20 s followed by an anodic potential scan from −1.0 to 1.3 V at a scan rate of 100 mV s^−1^ in 0.1 M KNO_3_ solution; (2) the uptake of Cd(II) was tested at different times (30, 60, 120, 180, and 300 s) at open circuit potential, under constant stirring in 1 × 10^−3^ M Cd(II) + 0.1 M KNO_3_ solution; (3) the electrode with pre-concentrated Cd(II) was transferred to a 0.1 M KNO_3_ solution free of Cd(II) to perform the DPV, which consisted of reducing the pre-concentrated Cd(II) to Cd(0) on the electrode surface at −0.95 V for 40 s, followed by anodic stripping (oxidation of Cd(0) to Cd(II)) from −0.95 to 0.6 V with a 33.3 mV s^−1^ scan rate, 40 mV pulse height, 75 ms pulse width, and 5 mV step height. The DPV conditions were optimized according to the voltametric response of the re-dissolved Cd(II) on a modified CPE electrode, which is observed in Supplementary Figure S1. The Supplementary Materials also describe the procedure for optimizing the accumulation-reduction potential. DPVs were obtained in a concentration range of 3–70 ppb. The voltametric responses of each concentration were used to estimate the electric charges associated with the corresponding anodic peak currents. The experimental responses were adjusted linearly to obtain a calibration curve for the electrode modified with PAA-co-IA/NaOH/MWCNTs. The LODs (limits of detection) and LOQs (limits of quantitation) of the calibration curve were calculated using Equations (2) and (3), where *s* is the standard error of the linear regression and *m* is the slope.

(2) $$LOD=\frac{3.3s}{m}$$

(3) $$LOQ=\frac{10s}{m}$$

## 3. Results and Discussion

### 3.1. Fourier-Transform Infrared Spectroscopy (FTIR)

**Figure 2:**
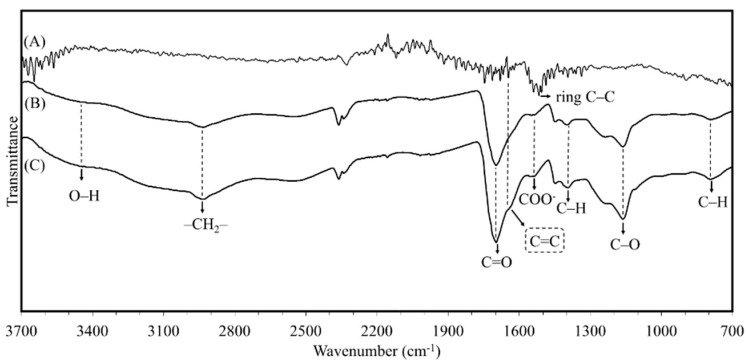
The infrared spectrum of (**A**) B-MWCNTs, (**B**) PA-co-IA/NaOH hydrogel, and (**C**) bamboo-like multiwall carbon nanotubes (PAA-co-IA/NaOH/B-MWCNTs).

Figure 2 shows the infrared spectra of the B-MWCNTs and PAA-co-IA/NaOH and the PAA-co-IA/NaOH/B-MWCNT hydrogels. B-MWCNT spectra (A) show the band associated with the ring C–C stretching at 1516 cm^−1^. The polymer spectra (B,C) show the band associated with the O–H stretching at 3450 cm^−1^, which is typical for PAA and similar polymers [34,35]. A band at 2937 cm^−1^ is consistent with the extension links –CH_2_– in the polymer chains, as well as the bands at 1394 and 795 cm^−1^, which indicate C–H bond deformations. On the other hand, it is possible to distinguish bands corresponding to the harmonics and combinations of bands near 1413 and 1248 cm^−1^ augmented by Fermi resonance [36] with the wide O–H stretching peak between 3660 and 2383 cm^−1^, as well as to identify C=O and C–O stretching bonds from carboxylic acid groups at 1697 and 1160 cm^−1^, respectively. The band at 1540 cm^−1^ corresponds to carboxylate groups. The spectrum of PAA-co-IA/NaOH/B-MWCNTs shows an additional band located at 1641 cm^−1^ associated with the C=C bond present in B-MWCNTs. These results confirm the effectiveness of the synthesis process. Additionally, the band around 2400 cm^−1^ corresponds to the stretching of carbon dioxide (O=C=O), suggesting its presence in the large quantity of pores in hydrogels.

### 3.2. Morphological Analysis

**Figure 3:**
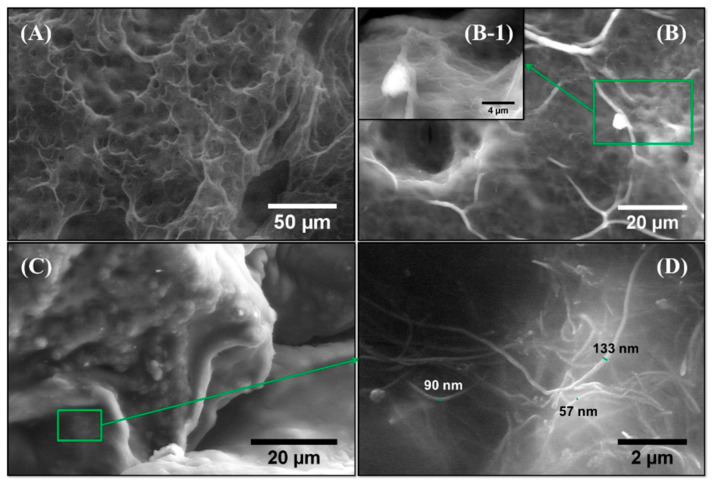
Scanning electron micrographs of freeze-dried hydrogels: (**A**) swollen PAA-co-IA/NaOH, (**B**) swollen PAA-co-IA/NaOH/B-MWCNTs (within an approach (**B-1**)), (**C**) dry PAA-co-IA/NaOH/B-MWCNTs, and (**D**) an enlargement of the area bounded by the rectangle.

The appearance of the dry and swollen PAA-co-IA/NaOH/B-MWCNT composite hydrogel is shown in Figure 1. The presence of the B-MWCNTs into the network of the swollen hydrogel can be clearly seen as a dispersion of small black agglomerates compared with the transparent gel. The agglomeration of B-MWCNTs was a result of their hydrophobicity since they were only partially oxidized in an air atmosphere (10% weight loss) [25]. The morphology and structure of the hydrogel composite were further studied by SEM, which allowed us to observe the three-dimensional network of the hydrogel, as well as the dispersion and thickness of the incorporated B-MWCNTs. Figure 3A shows the pure PAA-co-IA/NaOH hydrogel swollen with distilled water, which showed a highly porous content. Figure 3B shows the PAA-co-IA/B-MWCNT hydrogel with agglomerations and certain threads in parts of the network that are not observed in the polymer without nanotubes. In Figure 3B there is an enlargement of the image (Figure 3(B-1)) that shows the threads containing a relief that could form part of the nanotube encapsulation in the hydrogel. Figure 3C shows the dry composite hydrogel where the fading of the network can be observed, and an enlargement of an area (Figure 3D) clearly shows the threads corresponding to the carbon nanotubes with diameters ranging from 57 to 133 nm.

### 3.3. Water-Absorbing Capability

**Figure 4:**
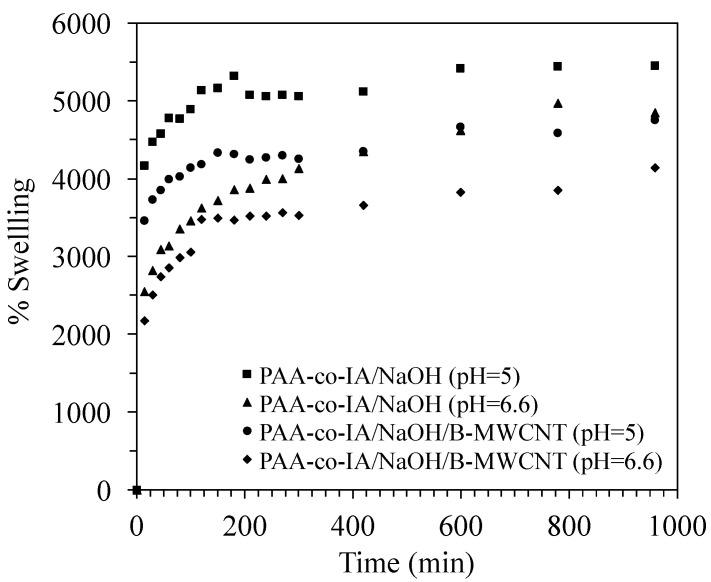
Swelling kinetics of pure (PAA-co-IA) and modified (PAA-co-IA/B-MWCNT) hydrogels in aqueous buffered solutions (pH = 5 and pH = 6.6) at room temperature.

The swelling capacity of pure PAA-co-IA/NaOH and modified PAA-co-IA/NaOH/B-MWCNT hydrogels was determined at pH 5 and 6.6. Figure 4 shows that both hydrogels had different swelling rates all the time and reached the plateau. In the carbon nanotube composite the swelling capacity decreased, since at pH 5 the PAA-co-IA/NaOH and PAA-co-IA/NaOH/B-MWCNT hydrogels reached swell abilities of 5450 and 4755%, respectively. This behavior can be attributed to the presence of B-MWCNTs, which can act as crosslinking agents during the polymerization, reducing hydrogel flexibility.

It is interesting to point out that the hydrogels developed in this work have shown a greater degree of swelling in comparison to some PAA/graphene oxide hydrogels developed by Bejarano-Jimenez et al. [32], which was attributed to the presence of itaconic acid in the polymeric chain as well as their neutralization rate. The overall effect of the copolymer was to increase the carboxyl groups available for ionization, which in turn increased the electrostatic repulsions between the polymeric chains, generating an expansion of the polymer network. However, at a higher pH (6.6) of the solution, the percentage of swelling decreased in comparison with the lower pH (5.0), suggesting that increases in the degree of ionization of carboxylic acid groups is accompanied by hydrogen bonding with water, which decreases the ionic pressure inside the hydrogel (deswelling) in a competitive mechanism of water diffusion in the hydrogel (swelling) [33,37]. In addition, similar swelling-pH decrements have been reported that suggest the change-screening effects of cations [38,39,40]. Nevertheless, more studies are necessary to confirm this. For this study, it was not necessary to carry out further tests at higher pH values since the hydrogel lost its elastic and mechanical properties. Neither were measurements carried out at pHs lower than 5 because the polymer was in its protonated form, which caused electrostatic repulsions of Cd(II), as mentioned later.

### 3.4. Thermal Properties of PAA-co-IA/NaOH/B-MWCNT Composite Hydrogel

**Figure 5:**
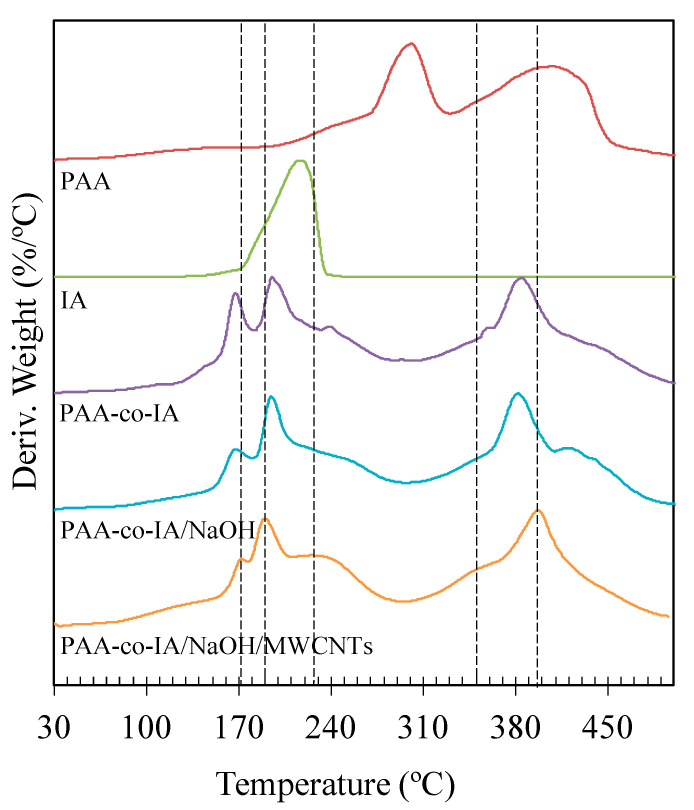
Derivative thermograms (DTG) of the PAA hydrogel, IA monomer, PAA-co-IA hydrogel, PAA-co-IA/NaOH hydrogel, and PAA-co-IA/NaOH/B-MWCNT hydrogel.

The thermal stability of the copolymer was determined through the derivative thermogravimetry (DTG) of individual components of hydrogels. Thermograms of PAA-co-IA/NaOH and PAA-co-IA/NaOH/MWCNT hydrogels, as well as a PAA-co-IA hydrogel without NaOH, are presented in Figure 5. As observed, poly(acrylic acid) (PAA) was degraded in two primary steps. The first breakdown peak (300 °C), corresponds to decarboxylation and the second (410 °C) to the depolymerization reaction. Both results are consistent with what was reported in the literature [41]. On the other hand, the thermograms of PAA-co-IA and PAA-co-IA/NaOH show significant differences due to IA decomposition, which are associated with the signal at 215 °C. The first peak around 167 °C of the copolymer indicates less thermal stability than PAA-co-IA/NaOH/B-MWCNTs, whose thermogram presents a shift of the peak to 173 °C. A second peak around 195 °C could be related to the decarboxylation of the second carboxylic group. The DTG thermogram of PAA-co-IA/NaOH/B-MWCNTs shows five decomposition temperatures: 173, 190, 226, 355, and 397 °C. The first two are coherent with the breakdown of PAA-co-IA with and without NaOH. Nevertheless, the shifts from 167 to 173 °C and 195 to 190 °C were influenced by the presence of B-MWCNTs, which affected the decarboxylation step. This suggests that the carbon nanotubes improved the crosslinking due to the increment of degradation temperature in the first peak, even with the decrease of thermostability in the second step. Different peaks at 226 and 355 °C also mark the influence of B-MWCNTs in thermal features. Also, the fifth peak presents a shift of 15 °C, from 382 to 397 °C, that corroborates the crosslinking between B-MWCNTs and carboxylic acid groups.

**Figure 6:**
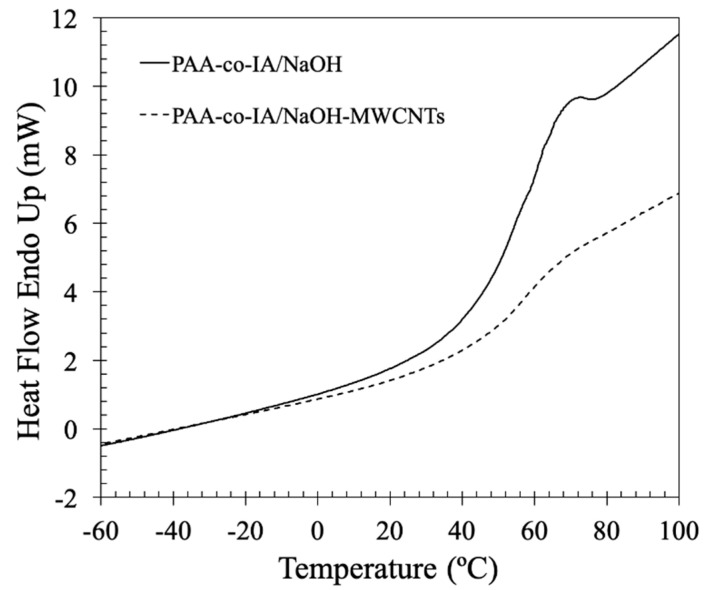
DSC thermograms of the PAA-co-IA and PAA-co-IA/NaOH hydrogels.

Figure 6 shows the thermogram of the DSC analysis. On one hand, it is observed that the insertion of B-MWCNTs to the polymeric matrix produced a small shift of 1 °C from 53 to 54 °C, indicating a better crosslinking. On the other hand, the same insertion resulted in a decrement of the delta Cp from 11.2 J/g°C (PAA-co-IA/NaOH) to 4.1 J/g°C (PAA-co-IA/NaOH/B-MWCNTs), which suggests the formation of chemical bonds between the B-MWCNTs and the hydrogel during the copolymerization reaction, which is consistent with the previous thermogravimetric analysis. In this context, it is known that materials with high crosslink densities present a decrease in heat capacity and Tg, making the transition difficult to identify [42].

### 3.5. Voltammetric Behavior of Cd(II) on CPE Modified with Hydrogels

The capacity of the carbon paste electrode modified with the PAA-co-IA/NaOH/B-MWCNT hydrogel to pre-concentrate the analyte was studied in a 1 × 10^−3^ M Cd^2+^ and 0.1 M KNO_3_ solution at pH 5. Supplementary Figure S2 shows the DPV response of cadmium after 2 min of accumulation under open circuit potential. The voltammogram presents an anodic peak around −0.8 V, which corresponds to the anodic stripping of cadmium. As a comparison, the voltammogram of the unmodified CPE shows a small broad peak, which is difficult to assign to a redox reaction associated with Cd(II) at the electrode surface. However, this small anodic peak provides evidence of the absence of a significant pre-concentration, and as a consequence limited diffusion of Cd(II) on the bare electrode, where the electron transfer will take place on the first layer of graphite [43]. On the other hand, the distinguished and remarkable enhancement of the oxidation peak of cadmium obtained with the modified electrode was due to the high surface area of the swollen PAA-co-IA/NaOH/B-MWCNT hydrogel, which possessed more active sites that could promote the adsorption and electron transfer between the electrode and analyte. Besides, the working pH 5 ensured that the cadmium in solution was in a cationic form, which could form coordination compounds with the carboxylate groups of the hydrogels [44]. Also, it has been reported that at this pH an acrylic acid-itaconic acid hydrogel can retain higher Cd(II) amounts than other M^2+^ such as Zn(II), Hg(II), and Ni(II) [31], and presents a greater degree of swelling as well. In this study, the point of zero charges of PAA-co-IA/NaOH and PAA-co-IA/NaOH/B-MWCNT hydrogels were measured at pH 3.7 and 4.7, respectively (see Supplementary Figure S3). Therefore, considering the working pH of the solution, it is feasible that the hydrogels possessed a negative surface charge to attract Cd(II) [32], which contributed to the enhancement of the anodic peak. This was confirmed by a test in solution at pH 3.5 where a decrease in the current of the anodic peak was observed, since the surface charge adopted by the polymer was positive and caused a repulsion of Cd(II).

Supplementary Figure S4A shows that the gradual increase in Cd(II) concentration resulted in a gradual increase of the anodic peak current around −0.85 V, which confirms that this peak corresponded to the oxidation of Cd(0) to Cd(II), and that the carbon paste electrode modified with PAA-co-IA/NaOH/B-MWCNTs was applicable for the detection of this analyte. Supplementary Figure S4B of the DPV response corresponding to the electrode modified with PAA-co-IA/NaOH hydrogel is also shown, for comparison.

### 3.6. Effect of Accumulation Time of Cd(II) in the PAA-co-IA/NaOH and PAA-co-IA/NaOH/B-MWCNT Hydrogels

**Figure 7:**
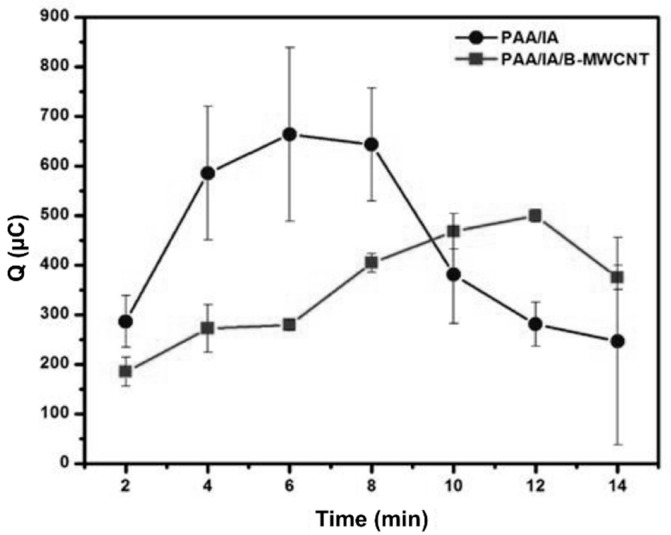
Effect of accumulation time on Cd(II) in CPE modified with either PAA-co-IA/NaOH (PAA/IA) or PAA-co-IA/NaOH/B-MWCNT (PAA/IA/B-MWCNT) hydrogel in a solution of Cd(II) 1 × 10^−3^ M + KNO_3_ 0.1 M at pH 5. Each point was obtained by DPV using the following conditions: The accumulation time was 2 min at open circuit potential under stirring, and the pre-concentrated Cd(II) was reduced to Cd(0) at −0.95 V for 40 s, followed by anodic stripping (oxidation of Cd(0) to Cd(II)) from −0.95 to 0.6 V with a 33.3 mV s^−1^ scan rate, 40 mV pulses height, 75 ms pulses width, and 5 mV step height. Each point was measured eight times in argon-saturated solutions.

The effect of accumulation time of Cd(II) on the surface of CPEs modified with either the PAA-co-IA/NaOH or PAA-co-IA/NaOH/B-MWCNT hydrogel was studied to assess the role of the B-MWCNTs in the polymeric network. The swelling capacity of the hydrogels on the CPEs was expected to have a significant influence on the voltametric response. Therefore, a study was carried out to establish the relationship between the voltametric signal and the accumulation time at the surface of the carbon paste electrode. Figure 7 shows the variations of the electric charge of the anodic stripping peaks of pre-concentrated Cd(II) on the modified CPE surface versus the immersion time in the solution of 1 × 10^−3^ M Cd(II) (i.e., 1.16 × 10^5^ ppb). A longer immersion time would result in an increase of the anodic charge (anodic current) until a maximum was reached. This maximum can be apparently explained by the equilibrium achieved between the Cd(II) bound to the modifier and the one, which is solubilized in solution [22]. The electric charge varied according to the modifier of the CPE. The PAA-co-IA/NaOH hydrogel reached its maximum storage capacity at 6 min, and after 8 min the load began to decrease, meaning that there were fewer carboxylate groups available to form coordination compounds with Cd(II). Also, at a time longer than 8 min, the detection signal changed constantly, probably due to the hydrogel content on the electrode surface beginning to fragment. Instead, the electrode modified with PAA-co-IA/NaOH/B-MWCNT achieved its maximum at 12 min, requiring a longer time to achieve a balance in the swelling test because the PAA-co-IA hydrogel at pH 5.0 absorbed more water than the PAA-co-IA/NaOH/B-MWCNT hydrogel. The last one presented less swelling capacity due to the presence of the B-MWCNTs, which probably acted as crosslinking agents, causing some restrictions to the water diffusion into the PAA-co-IA/NaOH/B-MWCNT hydrogel and thus slowing down the cadmium adsorption process, resulting in a longer time to reach equilibrium. As was demonstrated in the previous work by Bejarano-Jimenez et al. [32], the incorporation of the PAA/graphene oxide (GO) hydrogel as a modifier element of CPEs enhanced the detection of Cd(II) using DPV. The presence of GO enhanced the electrical signal of the electrodes in Cd(II) solutions. This property was influenced by the 10% and 20% degree of neutralization of the hydrogels. In this work, the CPE modified with either PAA-co-IA/NaOH or PAA-co-IA/NaOH/B-MWCNTs provided an anodic charge of 287.5 or 487.5 µC, respectively, after 12 min of accumulation (see Figure 7). Clearly, the PAA-co-IA/NaOH modifier had a greater adsorption capacity of Cd(II) in a shorter time, which could be attributed to a greater number of carboxylic groups in this hydrogel. In the case of the PAA-co-IA/NaOH/B-MWCNT hydrogel, the B-MWCNTs contained in its structure may behave as a crosslinking agent for polymer reducing swellability and increasing the flexibility of the material, as was observed for those hydrogels containing GO [45,46]. These aspects restricted the diffusion process of the analyte within the hydrogels, and thus the hydrogels required more time to be saturated.

On the other hand, at times longer than 12 min, both hydrogels began to fragment at the electrode surface, a situation that was undesirable since it may lead to poor reproducibility of readings. The swelling of the hydrogel causes the formation of structures like a cauliflower that can be seen with the naked eye on the surface of the electrode. Fragmentation was favored due to agitation during the pre-concentration of Cd(II) at open circuit potential and the fragments were seen at the bottom of the cell. In this test, the electrode modified with PAA-co-IA/NaOH/B-MWCNTs presented a significant reduction of the uncertainty of the quantification of Cd^2+^ due to the higher mechanical stability of this hydrogel, which contributed to better reproducibility of the voltametric signal. Therefore, the maximum value of time used for Cd(II) accumulation was 10 min to avoid the excessive swelling. An increase in the size of hydrogel particles on the electrode surface would cause hydrogel fragmentation, which may decrease the overall conductivity of the electrode surface and produce a degradation of Cd signals. These results confirm that accumulation time played a major role in the detection of Cd(II) when these hydrogels were used as modifying agents.

Concerning the B-MWCNTs, Pérez et al. [47] reported that these were able to remove Cd(II), suggesting that the adsorption of this analyte can occur as a transfer of external mass from the bulk solution to the nanotube surface, followed by intraparticle diffusion into the nanotube pores. Furthermore, the conglomerates of partially oxidized B-MWCNTs were dispersed into the polymeric matrix where they could provide active sites for Cd(II) adsorption. Additionally, the electrochemical properties of the B-MWCNTs were influenced by their curvature, degree of oxidation, and doping with nitrogen, in contrast with the flat geometry and electronic character of GO [48]. Based on the resulting data, the peak current of Cd(II) was found to increase linearly, as was demonstrated with the calibration study of the CPE modified with PAA-co-IA/NaOH/B-MWCNTs.

### 3.7. Calibration Plot, Limit of Detection and Reproducibility

**Figure 8:**
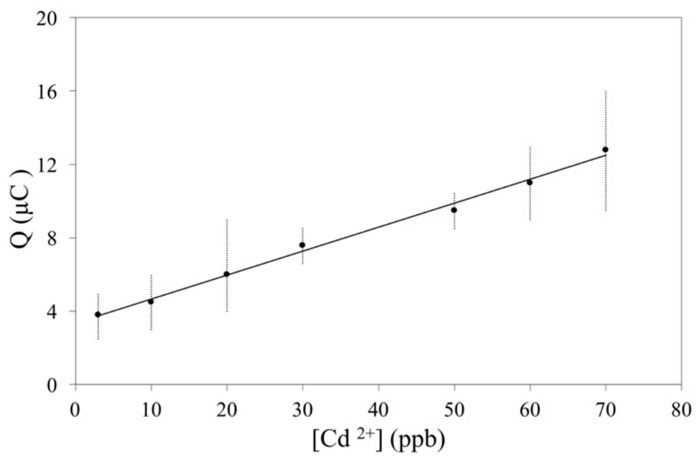
The calibration curve of Cd(II), at a concentration range of 2.67 × 10^−8^ and 6.23 × 10^−7^ M (i.e., 3 and 70 ppb) on the carbon paste electrode were modified with the PAA-co-IA/NaOH/B-MWCNT hydrogel in 0.1 M KNO_3_ argon-saturated solutions at pH 5. Each point was obtained by DPV using the following conditions: The accumulation time was 2 min at open circuit potential under stirring, and the pre-concentrated Cd(II) was reduced to Cd(0) at −0.95 V for 40 s, followed by anodic stripping (oxidation of Cd(0) to Cd(II)) from −0.95 to 0.6 V with a 33.3 mV s^−1^ scan rate, 40 mV pulses height, 75 ms pulses width, and 5 mV step height. Each point was measured eight times.

Figure 8 shows the calibration curve for the CPE modified with PAA-co-IA/NaOH/B-MWCNT in the standard solution of Cd^2+^. The calibration plot was found to be linear between 2.67 × 10^−8^ and 6.23 × 10^−7^ M (i.e., 3 and 70 ppb) with a slope of 0.15 µC ppb^−1^ L (R^2^ = 0.99) and an LOD and LOQ of 19.2 and 58.3 ppb, respectively. The time needed for preconcentration of Cd(II) in this case was longer, 10 min at OCP, since in this case the concentrations were lower than those used to obtain the voltametric responses shown in Supplementary Figures S2 and S4. This curve includes the maximum permissible limit of cadmium in water for human use and consumption, between 3 and 5 ppb [23]. Only with the electrode modified with the PAA-co-IA/NaOH/MWCNT hydrogel was it possible to detect cadmium(II) at 3 ppm, due to the mechanical resistance of the hydrogel provided by the B-MWCNTs as crosslinking points on one hand, and their partially oxidized surface offering additional active adsorption sites on the other.

In the Supplementary Materials it is shown that it was also possible to obtain two more linear calibration plots with this electrode at higher ranges of concentration as well as with shorter exposure times of the electrode in the solution. The composite hydrogel could nearly reach different adsorption equilibriums depending on the time of immersion as well as the concentration of Cd(II). Also, as mentioned above, the voltametric response was dependent on the swelling capacity of the hydrogel. Supplementary Figure S5A shows a linear calibration plot between 0.01 × 10^−3^ and 0.13 × 10^−3^ M (i.e., 1125 and 14,606 ppb) with a slope of 0.02 µC ppb^−1^ (R^2^ = 0.99) and a LOD and LOQ of 1800 and 5500 ppb, respectively. Both curves (Figure 8 and Supplementary Figure S5A) were obtained with an accumulation time of 10 min at open circuit potential.

With an increase in the concentration and a shorter accumulation time of 2 min, it was possible to obtain a linear relationship between concentrations of 0.9 × 10^−3^ and 2.1 × 10^−3^ M (i.e., 1.01 × 10^5^ and 2.36 × 10^5^ ppb) with a slope of 0.002 µC ppb^−1^ L (R^2^ = 0.99) (see Supplementary Figure S5B), showing that the increase of the Cd(II) concentration accelerated its pre-concentration while decreasing the sensibility of its detection. In order to evaluate the stability of the voltametric response of the modified CPE, each point of the calibration curves was tested eight times. The peak intensities changed due to the swelling and increase of the hydrogel particle size, which may not have been homogeneously dispersed on the electrode surface, causing variation of the relative standard deviation of the Cd signals for each concentration. However, the electrochemical method possessed favorable repeatability, which can be improved in further studies.

On the other hand, Supplementary Figure S5C shows the calibration curve for the CPE modified with PAA-co-IA/NaOH hydrogel. It was the only plot found to present a better fit to a straight line for a concentration range between 0.1 × 10^−3^ and 0.9 × 10^−3^ M (i.e., 1.1 × 10^4^ and 1.01 × 10^5^ ppb) with a slope of 0.001 µC ppb^−1^ L (R^2^ = 0.99), compared with the linear regression for other ranges of concentrations, meaning that this electrode was less sensitive and had less reproducibility than the electrode containing the PAA-co-IA/NaOH/B-MWCNT hydrogel. Further studies will consider the preparation of specific molecular weights of the polymers to improve the degree of uncertainty.

### 3.8. Interference Studies

**Figure 9:**
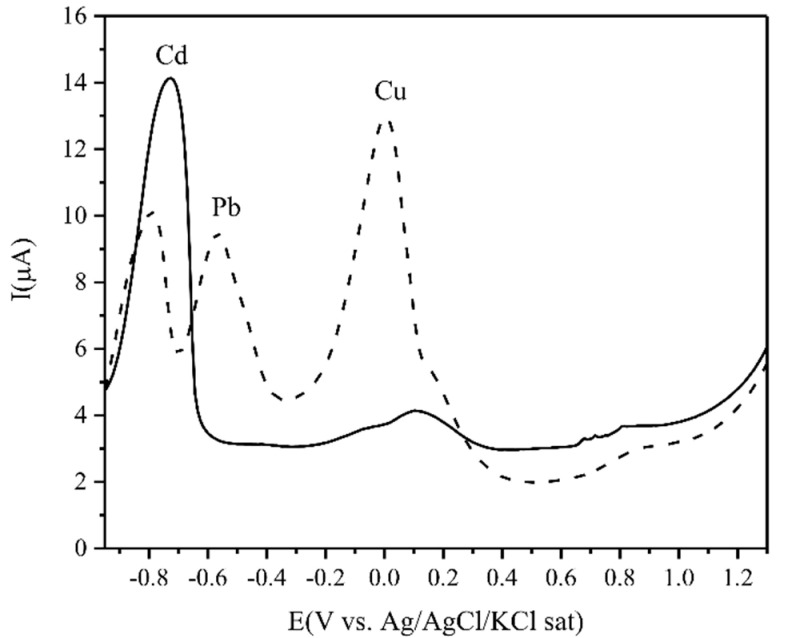
DPV of a mixture of other metal cations on CPE modified with PAA-co-IA/NaOH/B-MWCNT hydrogel in a solution of 3 ppb (2.67 × 10^−8^ M) Cd(II) (solid line) and 3 μgL^−1^ each of Cd(II), Zn(II), Hg(II), Pb(II), and Cu(II) (dot line) + 0.1 M KNO_3_ at pH 5. The accumulation time was 10 min at OCP under stirring, and the electrode with the pre-concentrated analytes was transferred to a solution free of these cations. The accumulated cations were reduced to their metallic form at −0.95 V for 40 s, followed by anodic stripping (oxidation of Cd(0) to Cd(II), Pb(0) to Pb(II), and Cu(0) to (Cu(II) from −0.95 to 0.6 V with 33.3 mV s^−1^ scan rate, 40 mV pulses height, 75 ms pulses width, and 5 mV step height. The solutions were saturated with argon at room temperature.

The interference of the voltametric response of Cd(II) was also studied in the presence of other metal cations, such as Pb(II), Zn(II), Hg(II), and Cu(II), which could be adsorbed simultaneously under the conditions for adsorption of Cd(II) [49]. Figure 9 displays the voltametric peak intensities in the presence and absence of these metal ions. It was observed that the Zn(II) and Hg(II) ions had no obvious influence on the voltametric peak intensities of Cd(II), and that the addition of Pb(II) and Cu(II) cations resulted in a decrease of the Cd(II) voltametric peak and the appearance of the Pb(II) and Cu(II) voltametric peaks. The metal ion concentration in this experiment was the same as the Cd(II) concentration, 3 ppb (i.e., 2.67 × 10^−8^ M), so it was evidently a competitive adsorption mechanism during the pre-concentration process, causing a recovery of 35% of the voltametric response of Cd(II) in the mixture of all the metal cations. It has been reported that the metal ion binding properties of linear hydrosoluble poly(acrylic acid) with 3 × 10^6^ of average molecular weight increased in the following order: Ni(II) < Cd(II) < Cu(II) < Pb(II) [41]. Also, as mentioned above, an acrylic acid-itaconic acid hydrogel presented a higher retention of Cd(II) than other M^2+^ such as Zn(II), Hg(II), and Ni(II) [31], which explains the greater accumulation of Pb(II) and Cu(II) in the hydrogel composite, and therefore their detection together with Cd(II).

Furthermore, other studies have proven that PAA composite films have great capacities to sense Cu(II) and Pb(II) [50,51] due to the binding of these cations with the carboxyl groups on PAA through electrostatic interaction. On the other hand, Kiani et al. [52] recently demonstrated the sensing of Zn(II) and Hg(II) by their interaction with amide groups instead of carboxyl groups, which has provided a different route for detection mechanism.

## 4. Conclusions

The polymeric material containing itaconic acid possessed an increased swelling capacity due to the presence of many carboxylate groups. The swelling capacity was affected by the presence of B-MWCNTs since they acted as crosslinking agents, decreasing the swelling capacity but providing greater mechanical resistance. The electrochemical detection of cadmium by the carbon paste electrodes modified with PAA-co-IA/NaOH/B-MWCNT hydrogel confirmed that such material can pre-concentrate Cd(II), and therefore provided electroanalytical signals derived from its re-oxidation by anodic stripping voltammetry techniques. The electrodes were able to detect 3 ppb of Cd(II) like those stipulated as the maximum permissible limit in water for human use and consumption (3 to 5 ppb). The modified electrode was able to detect Cd(II) in the presence of other metal cations, and therefore further studies to try to better understand the behavior of the composite in the presence of these metals are being carried out, which is very important for future applications. This composite material can be potentially applied for electrochemical detection of other water contaminants, such as disinfection by-products [53] and algal toxins [54,55,56].

## Supplementary Materials

The following are available online at https://www.mdpi.com/2079-6374/10/10/147/s1, Figure S1: Voltametric behavior of [Fe(CN)_6_]^3−^/[Fe(CN)_6_]^4−^ on CPEs modified with hydrogels; Figure S2: DPV response of Cd(II) in CPE modified with PAA-co-IA/NaOH/B-MWCNT hydrogel (solid line) and without modification (dot line) in solution of Cd(II) 1 × 10^−3^ M + KNO_3_ 0.1 M to a pulse reduction of −0.95 V during 40 s, 33.3 mVs^−1^ and pH 5, in argon saturated solutions; Figure S3: Estimation of the zero charge point of PAA-co-IA/NaOH and PAA-co-IA/NaOH/B-MWCNT hydrogels; Figures S4A and S4B: Voltametric behavior of Cd(II) on CPE modified with hydrogels adding different concentrations of the analyte; Figure S5: The calibration curves of Cd(II), at different concentration ranges on electrodes modified with PAA-co-IA/NaOH/B-MWCNT: A) 0.01 × 10^−3^ and 0.13 × 10^−3^ M (i.e., 1125 and 14,606 ppb); B) 0.9 × 10^−3^ and 2.1 × 10^−3^ M (i.e., 1.01 × 10^5^ and 2.36 × 10^5^ ppb); on electrode modified with PAA-co-IA/NaOH C) 0.1 × 10^−3^ and 0.9 × 10^−3^ M (i.e., 1.1 × 10^4^ and 1.01 × 10^5^ ppb).
