# Supplementary material for: Synthesis of Bamboo-like Multiwall Carbon Nanotube–Poly(Acrylic Acid-co-Itaconic Acid)/NaOH Composite Hydrogel and its Potential Application for Electrochemical Detection of Cadmium(II)
Source: Biosensors (Basel). 2020 Oct 19;10(10):147. doi: 10.3390/bios10100147 (PMC7603206; doi:10.3390/bios10100147)
Supplement: Supplementary file 1 [file biosensors-10-00147-s001.pdf]

biosensors

Supplementary information

A Bamboo-like Multiwall Carbon Nanotube–Poly(Acrylic Acid-co-Itaconic Acid)/NaOH Composite Hydrogel for Electrochemical Detection of Cadmium(II)

Luis F. Cházaro-Ruiz [1,*], Miguel Olvera-Sosa [2,3], Gabriela Vidal [1], J. René Rangel-Méndez [1], Gabriela Palestino [2,3], Fatima Perez [4] and Wei Zhang [5,*]

1 División de Ciencias Ambientales, Instituto Potosino de Investigación Científica y Tecnológica, A.C., Camino a la Presa San José 2055, Lomas 4ª Sección, San Luis Potosí C.P. 78216, SLP, México; f_ivanka@hotmail.com (G.V.); rene@ipicyt.edu.mx (J.R.R.-M.)
2 Facultad de Ciencias Químicas, Universidad Autónoma de San Luis Potosí, Manuel Nava No. 6, San Luis Potosí C.P. 78210, México; miguel_olvera@outlook.com (M.O.-S.); palestinogabriela@uaslp.mx (G.P.)
3 Sección de Biotecnología, Centro de Investigación en Ciencias de la Salud y Biomedicina, Universidad Autónoma de San Luis Potosí, Avenida Sierra Leona 550, Lomas Segunda Sección, San Luis Potosí C.P. 78210, México
4 CONACYT—Instituto Potosino de Investigación Científica y Tecnológica, A.C., Camino a la Presa San José 2055, Lomas 4ª Sección, San Luis Potosí C.P. 78216, SLP, México; fatima.perez@ipicyt.edu.mx
5 College of Engineering, Bay Campus, Swansea University, Swansea SA1 8EN, UK
* Correspondence: luis.chazaro@ipicyt.edu.mx (L.F.C.-R.); wei.zhang@swansea.ac.uk (W.Z.)

Voltammetric behavior of $[Fe(CN)_6]^{3-}/[Fe(CN)_6]^{4-}$ on CPEs modified with hydrogels.

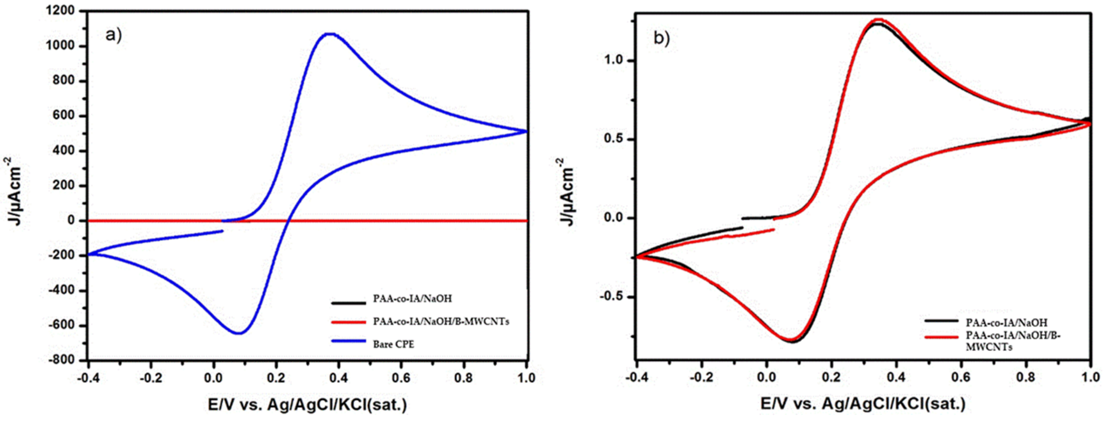

Figure S1. Cyclic voltammetric response of $[Fe(CN)_6]^{3-}/[Fe(CN)_6]^{4-}$ on carbon paste electrodes modified with PAA-co-IA/NaOH (black line) or PAA-co-IA/NaOH/B-MWCNTs (red line) hydrogel compared with the electrode without modification (blue line) in 0.01 M $[Fe(CN)_6]^{3-}/[Fe(CN)_6]^{4-}$ + 0.1 M KCl solution at pH 6, υ=20 mVs^{-1} scan rate, in argon saturated solutions at room temperature. The decrease in current of the modified electrode responses was due to electrostatic repulsion between the negative surface charge of the hydrogels and the electroactive anion.

Voltammetric behavior of Cd(II) on CPE modified with hydrogels.

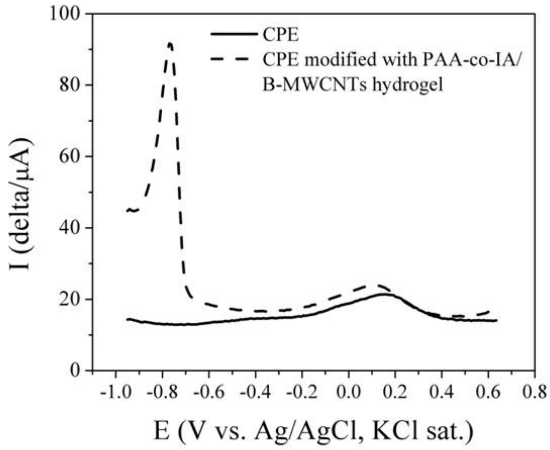

Figure S2. DPV response of Cd(II) preconcentrated on the surface of carbon paste electrode modified with PAA-co-IA/NaOH/B-MWCNTs hydrogel (solid line) compared with the electrode without modification (dot line) in 1 x 10^{-3} M Cd(II) + 0.1 M KNO_3 solution at pH 5. The accumulation time was 2 min at OCP under stirring. The electrode was transferred to a solution free of Cd(II) where the the pre-concentrated Cd(II) was reduced to Cd(0) at -0.95 V for 40 s, followed by anodic stripping (oxidation of Cd(0) to Cd(II)) from -0.95 to 0.6 V with 33.3 mV s^{-1} scan rate, 40 mV pulses height, 75 ms pulses width, 5 mV step height. The solutions were saturated with argon at room temperature.

Optimization of accumulation-reduction potential.

The influence of cathodic potential to reduce the pre-concentrated Cd(II) to Cd(0), on the intensity of anodic peak current associated with the re-oxidation of Cd(0) to Cd(II) was studied by varying the potential value from -0.85 to -1.0 V. When the potential was −0.95 V, a well-defined peak with the highest peak current was obtained (see Figure S1). A more negative reduction potential from -0.95 to -1.0 V led to the decrease of current peak. This was mainly due to the H_2 gas bubbles evolution at such negative potential, which could interfere with the process of accumulation of Cd(II). Hence, -0.95 V was employed as an optimum accumulation/reduction potential value for further studies.

Point of zero charge.

The point of zero charge (PZC) of the hydrogels was determined by mass titration. The preparation of the samples was performed as follows: different amounts of the hydrogels (0, 0.005, 0.01, 0.015, 0.02, 0.025, 0.03 and 0.035 g) in beakers were weighed, and then 10 mL of deionized water, previously deoxygenated with argon, were added to each sample. The samples were kept at room temperature for 48 hours; once that time has elapsed the pH of the solution was recorded. The PZC was determined at the point at which the pH remained unchanged on a graphical variation of pH *vs.* the mass of the solid.

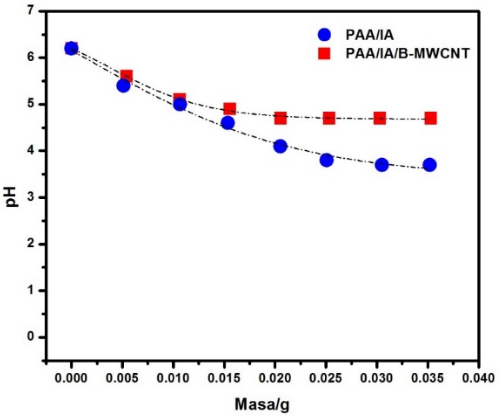

Figure S3. Estimation of the zero-charge point of PAA-co-IA/NaOH and PAA-co-IA/NaOH/B-MWCNTs hydrogels in aqueous batch argon saturated solutions at room temperature.

Voltammetric behavior of Cd(II) on CPE modified with hydrogels adding different concentrations of the analyte.

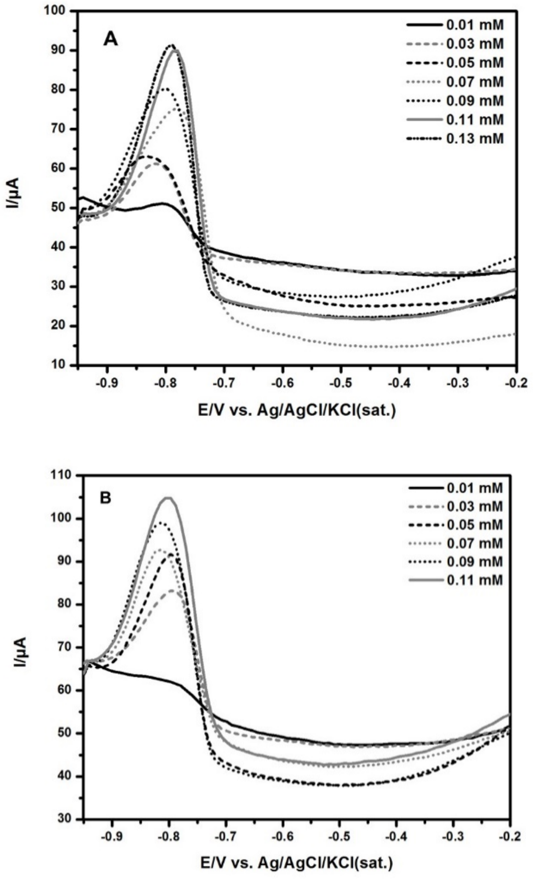

Figure S4. DPV responses corresponded to the gradual increase of Cd(II) concentration. The Cd(II) was pre-concentrated on the surface of carbon paste electrode modified with either A) PAA-co-IA/NaOH/B-MWCNTs or B) PAA-co-IA/NaOH hydrogel in (from $0.1x10^{-3}$ to $0.9x10^{-3}$ M) Cd(II) + 0.1 M KNO_3 solution at pH 5. The accumulation time was 2 min at OCP under stirring, and the electrode with the pre-concentrated analyte was transferred to a solution free of Cd(II). The accumulated Cd(II) was reduced to Cd(0) at -0.95 V for 40 s, followed by anodic stripping (oxidation of Cd(0) to Cd(II)) from -0.95 to 0.6 V with 33.3 mV s^{-1} scan rate, 40 mV pulses height, 75 ms pulses width, 5 mV step height. The solutions were saturated with argon at room temperature.

Calibration curves at higher concentrations of Cd(II) and shorter accumulation time on modified electrodes with PAA-co-IA/NaOH/MWCNTs (A) and (B), compared with the electrode modified with PAA-co-IA/NaOH (C).

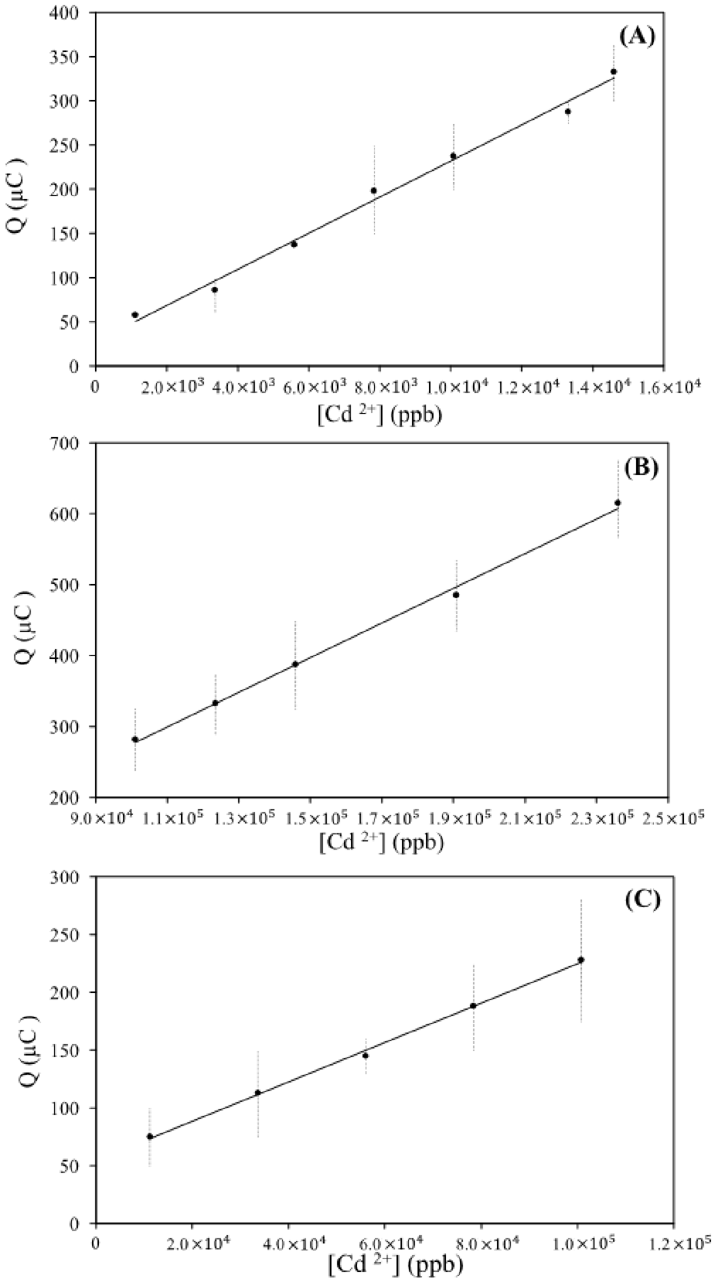

Figure S5. The calibration curves of Cd(II), at different concentration ranges: A) $0.01x10^{-3}$ and $0.13x10^{-3}$ M (i.e. 1,125 and 14,606 ppb); B) $0.9x10^{-3}$ and $2.1x10^{-3}$ M (i.e. $1.01x10^{5}$ and $2.36x10^{5}$ ppb) on the electrode modified with PAA-co-IA/NaOH/MWCNTs. The calibration curve C) obtained with a concentration range between $0.1x10^{-3}$ and $0.9x10^{-3}$ M (i.e. $1.1x10^{4}$ and $1.01x10^{5}$ ppb) on the electrode modified with PAA-co-IA/NaOH. The voltammetric experiment was performed in 0.1 M KNO_3 solution at pH 5. The accumulation time was 2 min at OCP under stirring, and the electrode with the pre-concentrated analyte was transferred to a solution free of Cd(II). The accumulated Cd(II) was reduced to Cd(0) at -0.95 V for 40 s, followed by anodic stripping (oxidation of Cd(0) to Cd(II)) from -0.95 to 0.6 V with 33.3 mV s^{-1} scan rate, 40 mV pulses height, 75 ms pulses width, 5 mV step height . The solutions were saturated with argon at room temperature.
